# 2335: Delayed rewarming for neuroprotection in infants following congenital heart surgery: A safety study

**DOI:** 10.1017/cts.2017.131

**Published:** 2018-05-10

**Authors:** Alexa Kanwit Craig

## Abstract

OBJECTIVES/SPECIFIC AIMS: Congenital heart disease (CHD) is the most frequently occurring birth defect in the United States affecting about 40,000 infants born every year. Despite significant advances in postsurgical survival, developmental outcomes remain disproportionately poor. Therapeutic hypothermia has been used for neuroprotection during cardiac surgery since the 1950s. Infants undergoing cardiac surgery are typically cooled to 28–33°C during the operation and then rapidly rewarmed to normothermia following surgery at a rate of 1°C every 3–5 minutes to minimize concerns surrounding the risks associated with prolonged bypass exposure. However, emerging evidence from animal models has shown rapid temperature changes following surgery may diminish or even negate the neuroprotective effect of intraoperative hypothermia. No prospective studies have assessed the safety or impact of alternative approaches to postoperative temperature management on the outcome of infants with CHD undergoing cardiac surgery. Therefore, we conducted a pilot study to examine the safety of a novel application of a temperature-regulating device to slowly rewarm infants with congenital heart disease over the 12 hours following cardiac surgery. METHODS/STUDY POPULATION: From November 2014 to July 2016, infants with CHD requiring surgery with cardiopulmonary bypass before the age of 12 months were prospectively recruited. Infants were randomized in blocks of 3 with 1 allocated to standard of care and 2 to the experimental protocol. Infants assigned to the standard of care were rewarmed in the operating room while on bypass at a rate of 1°C every 3–5 minutes back to a temperature of 37°C. Infants assigned to the experimental intervention, were rewarmed on bypass to 35°C and then over the subsequent 12 hours following surgery, gradually rewarmed using an FDA approved “cooling blanket” to increase temperature by 0.3°C every 2 hours for 6 hours and then by 0.2°C every 2 hours for 6 hours until the goal temperature of 36.5°C was achieved. Frequency of serious, moderate and other adverse events were tracked. Detailed vital sign data was collected hourly for the first 12 hours after surgery and then every 6 hours for the next 36 hours and included temperature, highest and lowest heart rate, highest and lowest systolic blood pressure, and highest and lowest diastolic blood pressure. Presence or absence of abnormal cardiac rhythms was recorded per 24-hour interval. Chest tube output was recorded in cc/kg/8 hours for as long as the chest tube was in place. Laboratory data points included serum creatinine level, serum glucose level, liver function tests (AST and ALT), platelet count, hematocrit level, PTT, INR, fibrinogen, white blood cell count and lactate. Blood samples for biomarkers of brain injury (s100b and NSE) were obtained on all infants at the following 4 intervals; the preoperative setting for baseline, postoperatively after bypass, on postoperative day 1, and on postoperative day 2. For this safety study, the primary outcome measure was a composite outcome of the frequency of serious adverse events as well as the frequency of any adverse events and was compared among treatment groups. Data were analyzed using an intent to treat analysis. The study was approved by the Maine Medical Center Institutional Review Board. RESULTS/ANTICIPATED RESULTS: Seven infants were randomized to the standard of care group and 9 were randomized to the experimental group. There were 2 exclusions after randomization in the standard of care group with 1 death in the operating room and 1 unsuccessful attempt to wean from bypass. The mean temperature upon arrival to the PICU for the experimental infants was 35.2°C (range 34–36°C) and for the standard of care infants was 37.5°C (range 36.9–38.9°C). For the first 8 hours after surgery, infants in the standard of care group had mean temperatures over 37.0°C. There were no significant differences in the frequency of serious, moderate, or other adverse events between the standard of care group and experimental group. No infant in either group had need for cardiopulmonary resuscitation or exploratory surgery within 48 hours following surgery nor did any infant experience any clinically appreciated adverse neurological events such as stroke or seizure. No infant in either group experienced clinically significant bradycardia of less than 100 beats per minute or sustained tachycardia of greater than 160 beats per minute. There was a trend toward lower heart rates in the experimental group. Junctional Ectopic tachycardia (JET) occurred in 2 patients in the experimental group and 1 in the standard of care group. The mean highest INR in both groups was 1.4 (range 1.2–1.6). The mean lowest recorded platelet level in the first 48 hours was 128.8 (range 87–160) in the standard of care group and 123.8 (range 49–229) in the experimental group. Infants in the experimental group had lower chest tube output overall than the standard of care infants. The mean days of intubation for standard of care infants was 5 days (range 1–15 days) and for experimental infants the mean was 3.7 days (range 0–16 d). The PICU length of stay was shorter for the experimental infants (6.9 vs. 12 d for standard of care). The total length of stay was also shorter for experimental infants (12.4 vs. 16.4 d for standard of care). Serum biomarkers of brain injury (s100b and Neuron specific enolase) were elevated in the immediate postoperative period for infants in the standard of care group compared with the experimental group but normalized more quickly for standard of care. DISCUSSION/SIGNIFICANCE OF IMPACT: This small pilot study suggests that mild hypothermia following congenital heart surgery in infants under the age of 12 months is safe as there was no increase in the rate of severe, moderate, or other adverse outcomes in infants who received the experimental treatment of delayed rewarming. This study provides evidence for the efficacy of the cooling blanket in regulating the temperature of infants after surgery. Trends toward lower chest tube output, shorter intubation and decreased length of stay are possibly the result of improved hemodynamic stability in the absence of postoperative fever. Future studies will need to assess the effect of mild hypothermia compared with a normothemic control group.

